# Local Ties, Trans-Local Ties, and Substance Use among Rural-to-Urban Migrants in China

**DOI:** 10.3390/ijerph19074233

**Published:** 2022-04-01

**Authors:** Xi Chen, Hua Zhong, Serena Yunran Zhang

**Affiliations:** 1 The Jockey Club School of Public Health and Primary Care, The Chinese University of Hong Kong, Hong Kong, China; chenxi424@gmail.com; 2 Department of Sociology, The Chinese University of Hong Kong, Hong Kong, China; yunranzhang@link.cuhk.edu.hk

**Keywords:** local ties, trans-local ties, alcohol use, tobacco use, rural-to-urban migrants, China

## Abstract

China has witnessed unprecedented rural-to-urban migration since the early 1980s. While trying to assimilate into the city, rural-to-urban migrants still maintain close ties with their home communities. This study examines how local ties and trans-local ties of rural-to-urban migrants affect their alcohol and tobacco use. Data were obtained from the 2016 and 2018 China Labor-force Dynamics Survey, a nationally representative sample of adults aged over 15 in 29 provinces in China. Participants included 1426 rural-to-urban migrant workers and 6438 urban residents in China. We found that compared to urban natives, rural-to-urban migrants had higher tobacco use prevalence (logit = 0.19, 95% CI = [0.03, 0.35]; *p* < 0.05) and more frequent alcohol use (logit = 0.27, 95% CI = [0.11, 0.42]; *p* < 0.001) after adjusting for sociodemographic characteristics. Migrants with more local social ties engaged in more frequent drinking (having >10 local friends vs. having 0 local friends: logit = 0.58, [0.10, 1.06], *p* < 0.05), whereas trans-local ties were not a significant correlate. In contrast, migrants who returned to their hometown more times (an indicator of trans-local ties) were more likely to be current tobacco users (logit = 0.01, 95% CI = [0.00, 0.02], *p* < 0.01) after adjusting for sociodemographic variables. These findings extended the research on social networks and health behaviors by identifying how local and trans-local ties differentially affected the vulnerabilities of tobacco and alcohol use among rural-to-urban migrants in China. The findings suggested that policies and interventions on reducing migrants’ health risk behaviors should focus on the role of different types of social ties.

## 1. Introduction

### 1.1. Tobacco and Alcohol Use among Rural-to-Urban Migrants in China

**Figure 1:**
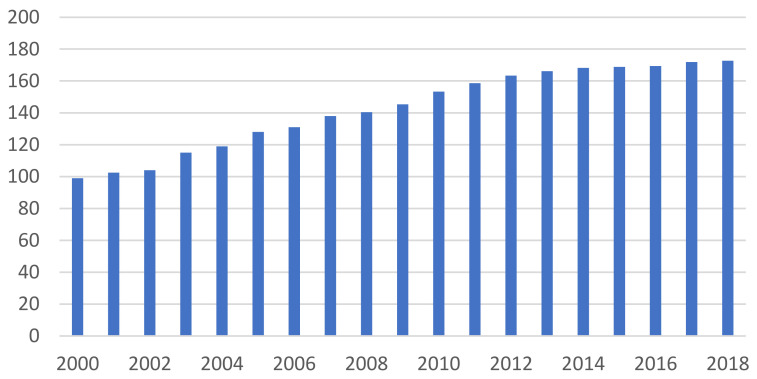
The rising number of rural migrants who left their rural hometown from 2000 to 2018 (million). Source: National Bureau of Statistics, China.

Although Western developed countries have completed urbanization in a much earlier historical period, there is large-scale ongoing rural-to-urban migration in most of the developing countries. The effects of such internal migration on migrants’ well-being have aroused substantial attention from researchers. As a typical developing society, China has witnessed unprecedented rural-to-urban migration along with its rapid modernization and urbanization process since the early 1980s. In the 1990s, China gradually became the “World Factory”, and brought a soaring demand for rural-to-urban workers; such demand continued in the 21st century [1]. Figure 1 shows that the number of rural migrant workers in China kept increasing and reached 173 million in 2018, a population even larger than the entire working population in the United States. Compared with rural-to-urban migrants in other developing countries, these Chinese migrant workers might be more vulnerable since they have been regarded as second-class citizens in their own country [2]. Although rural migrants are allowed to work in Chinese cities, they face difficulty in changing their official residency status from “rural” to “urban” due to the household registration (*hukou*) system [3]. *Hukou* is a discriminatory institutional arrangement in China established in the late 1950s to distinguish “urban” (non-agriculture) and “rural” (agriculture) residents so that urban residents have priorities in resource competition. For instance, the urban welfare system in China is more comprehensive and more generous than the rural one. Holding a “rural” *hukou* restricts rural migrants’ employment opportunities in a better-paid labor market, excludes them from the urban housing benefits, limits their rights to enjoy urban medical care and a pension, and causes identity discrimination against them [4,5,6,7]. Previous studies have documented a high level of psychological and social stress among rural-to-urban migrants in China due to their lower socioeconomic status (SES), unstable living and employment conditions, and insufficient social protection [8,9]. Such mental stress may result in elevated levels of health risk behaviors, particularly increasing their susceptibility to substance use as a way to cope with the stress [10,11].

Both tobacco and alcohol use are prevalent among Chinese rural-to-urban migrants. A meta-analysis in 2016 showed that the prevalence of tobacco use among Chinese internal migrants ranged from 17.03% to 41.42% (for studies including both female and male migrants), and the pooled prevalence of tobacco use was 27.75% (46.71% for males and 5.34% for females) [12]. Alcohol misuse was also reported to increase among rural-to-urban migrants [13]. A study of 2153 young rural-to-urban migrants found that about one-third (34.6%) of participants had experienced alcohol intoxication at least once during the past month [14]. However, the results were mixed regarding whether rural-to-urban migrants engage in higher levels of substance use than their urban counterparts. Several studies found that the prevalence of tobacco use is greater among rural-to-urban migrants than urban residents [10,15,16], whereas others have found that the prevalence of tobacco use was lower than that of urban residents [17,18,19]. Such inconsistent findings may be partly due to the variations in the selection of samples and control groups. National-level representative data are thus needed to ascertain whether rural-to-urban migrants engage in higher levels of substance use than urban residents in China.

### 1.2. Social Ties and Immigrants’ Health Behaviors

Previous studies on substance use among internal migrants in China mostly focus on their low SES and psychological distress as risk factors (e.g., [10,11]). However, substance use, as well as migration, are not only an individual choice but a network-based behavior, which are subject to interpersonal influence. Social ties may have a dualistic effect on health by either preventing or promoting health risk behaviors through psychological and behavioral mechanisms. On the positive side, social ties may generate social support that can help buffer stress, affect appraisals of stress, and alter the perceived capacity to cope [20]. Social connectedness can also create a sense of belonging and a positive psychological state that produce positive physiological responses and promote positive health behaviors [20]. On the negative side, however, social connections can expose individuals to environmental cues of health risk behaviors and possibly boost their engagement of risk behaviors due to behavioral contagion [21,22]. Additionally, social ties may produce psychological stress when social interactions involve excessive demands, restrict freedom, and require group conformity [23], which may, in turn, increase health risk behaviors.

Prior studies on international immigrants have identified that disrupted social networks and social isolation following migration may lead to health risk behaviors among immigrants [24,25]. However, these immigrants may maintain connections with home countries through transnational ties while at the same time building social ties in the receiving society. To date, only a few studies have examined how locations of social ties may affect substance use among immigrants. A study of Latino immigrants in the US found that greater perceived neighborhood social cohesion (local neighborhood ties) was associated with a decreased possibility of being a current smoker; however, the number of past-year return visits to the country-of-origin (transnational ties) was positively associated with their current smoker status [26]. Another study on Latino and Asian origin immigrants in the US found that cross-border ties were associated with a greater likelihood of past-year alcohol use for Latina women, whereas such ties were related to lower probabilities of past-year alcohol use among Asian immigrants [27]. Such findings highlight the importance of differentiating the locality of social ties when considering how ties to migrants’ host society and ties to the sending areas may affect their health behaviors. Beyond the international migration milieu, what has yet to be considered is whether trans-local ties matter in the internal migration setting.

### 1.3. Local Ties, Trans-Local Ties, and Health of Rural-to-Urban Migrants in China

Despite some relaxation of the *hukou* system in recent years, it remains difficult for rural-to-urban migrant workers in China to obtain an urban *hukou* and settle in the city permanently. Most rural migrants still engage in high circular mobility between urban areas and rural communities and sustain close ties with their home communities [9]. Moreover, taking advantage of the low-cost internet and communication technology, rural migrants in cities can keep social, emotional, and economic ties with their family and friends in the home community easily.

Some studies have examined the effect of local ties and trans-local ties on Chinese internal migrants’ health, focusing exclusively on their psychological well-being. Jin et al.’s (2012) [9] survey of migrant adults in Shanghai found that more trans-local ties were associated with better mental health among rural migrants, whereas the number of local ties was not a significant correlate of their mental health. The beneficial role of trans-local ties in migrants’ psychological well-being may be due to the social support and positive social comparison generated from the trans-local ties. Compared to local ties, trans-local ties tend to strengthen migrants’ social comparison with residents in the rural communities from which they migrate and can produce a more favorable evaluation of their social status as they generally make more money in cities. Similarly, research on rural-to-urban migrant children in China found that both local ties and trans-local ties enhanced migrant adolescents’ mental well-being, with trans-local ties being somewhat more useful in moderating social stress they experienced [28,29]. In contrast, other studies have identified certain negative or nonsignificant effects of local ties on migrant workers’ victimization and life satisfaction since their close interactions with urban natives may increase their risky lifestyles (e.g., more entertainment activities at night) or experience more discriminations from urban natives [6,30]; Yue et al.’s (2019) also pointed out the nonsignificant relationship between trans-local ties and migrant workers’ life satisfaction since trans-local contacts are also socially vulnerable and could not provide the help and support that migrants need [31].

Despite emerging evidence of the effect of local and trans-local ties on migrants’ health in China, two research gaps remain in the past literature. First, to the best of our knowledge, no studies have examined how local and trans-local ties may affect health risk behaviors among rural-to-urban migrants in China. Second, previous measures of the trans-local relations of rural-to-urban migrants in China either used the number of close friends and relatives outside the host cities [9] or the frequency of contact with home friends and relatives [28,29], which did not directly assess the social and economic activities of migrants in the home communities. We address that gap in this study by gauging migrants’ trans-local ties with the number of return visits, their economic contribution to rural communities where they migrated, and the help they offered to village fellows in their sending areas.

### 1.4. The Present Study

Based on the national-representative China Labor-Force Dynamics Survey (2016 and 2018), this study aims to examine the prevalence and correlated social factors of tobacco and alcohol use among rural-to-urban migrant workers in China, with a special focus on their social ties to host cities and to home communities. Although we target rural-to-urban migrants, we also include a sample of native urban residents for comparison, which may help us identify the unique features of substance use and the effects of social ties among migrants. We have two specific research questions in this study: (1) to explore whether rural-to-urban migrants in China have higher levels of tobacco and alcohol use than their urban counterparts, after adjusting for sociodemographic variables; and (2) to analyze how local and trans-local ties affect rural-to-urban migrants’ tobacco and alcohol use in China. Due to the inconsistent evidence of the prevalence of substance use among rural migrants in China and virtually nonexistent research on the effects of local and trans-local ties on their health risk behaviors, we raised research questions instead of proposing specific hypotheses. We organize the paper as follows: first, we outline the research methods and analytical strategies; we then describe the main results and discuss their theoretical and policy implications.

## 2. Materials and Methods

### 2.1. Data

This study used data from China Labor-force Dynamics Survey (CLDS), a nationwide multiple cross-sectional survey conducted biannually by the Sun Yat-Sen University in China since 2012 [32]. With a focus on labor force issues, the CLDS is a nationally representative study of individuals aged over 15, families, and communities in urban and rural areas across 29 of the 31 provinces of mainland China (excluding Tibet and Hainan). The survey applied multistage cluster proportionate probability sampling methods. The 29 provinces were first classified into six strata based on population size and geographic location. Rural counties, county-level cities, and county-level urban districts (primary sampling units, PSUs) within each stratum were then randomly selected according to their GDP rankings. The quantity of PSUs in each geographic stratum was determined by the size of their labor force. Within each PSU, urban and rural communities (secondary sampling units, SSUs) were randomly drawn based on their GDP rankings and the proportion of the internal migrant population. Finally, the households were selected at random from each SSU, and all family members aged over 15 were sampled. Detailed information of the CLDS data was shown elsewhere (e.g., [33,34]).

To reflect the recent pattern of substance use among rural-to-urban migrants in China, we analyzed the latest two waves of data available that were collected in 2016 and 2018. The 2016 CLDS included 7767 respondents in urban districts, among which 1234 were rural-to-urban migrants. The 2018 CLDS contained 4924 respondents in urban districts and 714 of them were rural-to-urban migrants. As the present study focused only on the working population, respondents who reported being unemployed since the last year were excluded from the analysis (n = 4827). The final sample size was 7864 respondents, including 1426 rural-to-urban migrants (without an urban *hukou*) and 6438 urban native residents (with an urban *hukou*).

### 2.2. Measurement

#### 2.2.1. Dependent Variables

*Tobacco Use*. The participants were first asked whether they have a tobacco use history (“Have you smoked one or more cigarettes per day for at least one year?”). For those who had a tobacco use history, they were further asked whether they were still smoking cigarettes currently (“Have you quit smoking or do you still smoke cigarettes currently?”). The tobacco use in our study thus was recoded as a binary variable (0 = current non-smoker; 1 = respondents with a tobacco use history and are currently using tobacco).

*Alcohol Use.* It was gauged by first asking the respondents whether they engaged in drinking alcohol (at least once a week). Respondents who reported “yes” to this question were further asked to report the frequency of drinking. The variable of drinking behavior was an ordinal variable that included four categories of response (1 = never/less than once a week, 2 = 1–2 times per week, 3 = 3–4 times per week, and 4 = almost every day).

#### 2.2.2. Independent Variables

*Local social ties* were measured by asking respondents to indicate the number of local friends they had in cities. We categorized the responses into four categories, i.e., no local friends, 1–5 local friends, 6–10 local friends, and more than 10 local friends.

*Local neighborhood cohesion* was assessed by three questions about perceptions of neighborhood support and trust: (1) “Do you know your neighbors?” (1 = very little; 5 = very well); (2) “Do you trust your neighbors?” (1 = very little; 5 = very much); and (3) “Is there mutual support between you and your neighbors and other residents in this urban community?” (1 = very little; 5 = very much). The mean of the three items was calculated, with higher scores representing a stronger perception of neighborhood cohesion (three-item scale, alpha = 0.82).

*Trans-local ties* were measured by three questions. The first question asked respondents about the number of times that they went back to their home communities in the past year. The second question asked respondents whether they had donated money to their home communities. The third question asked respondents whether they had helped people in their home communities.

#### 2.2.3. Control Variables

The control variables included age, sex (male vs. female), marital status (single, cohabitate, married, and divorced/widowed), education (primary school or below, secondary school, and college or above), occupation (manufacture industry, service industry, and other), logged annual income, and the region (East, Middle, and West).

### 2.3. Analytical Strategies

We first conducted descriptive analysis for all variables among the full sample and subsamples of rural-to-urban migrants and urban native residents. We also tested the differences in these variables between migrants and native urban residents using two-sided *t*-tests or *chi*-square tests. Next, we performed logistic regression to examine the effect of migration status on tobacco use and used ordinal logistic regression to estimate the effect of migration status on the frequency of drinking after controlling for other sociodemographic variables. We then investigated the associations between sociodemographic characteristics and tobacco and alcohol use among migrants and urban native residents using either logistic regression (for tobacco use) or ordinal logistic regression (for alcohol use) models. Lastly, we ran appropriate regression models to examine the effect of local ties and trans-local ties on tobacco and alcohol use among rural-to-urban migrants and native urban residents. We performed a formal test of multicollinearity. The variance inflation factor for each of the predictors was well below 10 (ranged from 1.02 to 2.36); thus, there were no extreme interrelations between predictor variables. All analyses were performed in Stata 16.0. The coefficients with 95% confidence intervals were reported. A *p* value of 0.05 was set as the level of statistical significance.

## 3. Results

### 3.1. Sample Characteristics

The descriptive statistics are displayed in Table 1. The mean age of the full sample was 43 (SD = 11.33). There were slightly more males (53.08%) than females (46.92%). A majority of respondents were married (81.37%), had secondary education (52.74%) or above (34.92%), and worked in the service industry (51.04%). More than half of the respondents were in East China (53.62%), with the remaining in the Middle (21.43%) and the West (24.95%). The comparisons between rural-to-urban migrants and local workers showed that migrant workers were younger, lower-educated, more likely to be single, more likely to work in the manufacturing industry, and more likely to be located in the East. There were no significant differences in sex and annual income between the two samples.

### 3.2. Differences in Local/Trans-Local Ties and Substance Use between Rural-to-Urban Migrants and Urban Natives

Nearly one in four respondents (24.42%) in the full sample were current smokers, with rural-to-urban migrants being significantly more likely to report current smoker status than local workers (26.95% vs. 23.86%, *p* < 0.05). About 80% (78.44%) of respondents never drank or drank less than once per week. Rural-to-urban migrant workers tended to drink more frequently than their urban counterparts. As for local ties, rural-to-urban migrants seemed to report more friends in the city than urban native residents. However, urban residents perceived a significantly higher level of neighborhood cohesion than migrants. On average, rural-to-urban migrants went back to their hometown about 5.6 times in the past year. Nearly one in four rural-to-urban migrants (23.29%) donated money to their home communities, and more than one-third of migrants (37.48%) helped people in the home communities. 

### 3.3. Associations between Migration Status and Substance Use

Table 2 presents the effect of migration status on tobacco and alcohol use. The results revealed that being a rural-to-urban migrant was positively associated with both tobacco use (logit = 0.19, 95% CI = [0.03, 0.35]; *p* < 0.05) and alcohol use (logit = 0.27, 95% CI = [0.11, 0.42]; *p* < 0.001), after controlling for other sociodemographic variables.

### 3.4. Associations between Background Variables and Substance Use

Table 3 shows the associations between sociodemographic variables and tobacco and alcohol use. Results in Model 1a revealed that among the rural-to-urban migrant workers, female sex (b = −4.51, 95% CI = [−5.24, −3.79]; *p* < 0.001) and college education or above (b = −0.62, 95% CI = [−1.18, −0.06]; *p* < 0.05) were associated with lower likelihood of tobacco use. The same pattern held for urban native workers (Model 1b). Migrant workers who were employed in the service sector were more likely to use tobacco (b = 0.53, 95% CI = [0.09, 0.97]; *p* < 0.05). However, urban residents who worked in the manufacturing industry were more likely to report tobacco use (b = 0.25, 95% CI = [0.06, 0.44]; *p* < 0.01). As for the alcohol use, Models 2a and 2b showed that females report less frequency of drinking among rural-to-urban migrants and urban native residents. Urban residents with higher annual income and who lived in the West tend to have a higher frequency of drinking, whereas socioeconomic variables were not significantly associated with alcohol use among rural-to-urban migrant workers.

### 3.5. Associations between Local/Trans-Local Ties and Substance Use among Rural-to-Urban Migrants and Urban Natives

The effects of local/trans-local ties on tobacco use were shown in Table 4. Model 3a demonstrated no significant association between the strength of local ties and tobacco use among the rural-to-urban migrant sample. In contrast, migrants who had more return visits to sending areas were more likely to use tobacco (Model 3b: b = 0.01, 95% CI = [0.00, 0.02]; *p* < 0.01). Model 4 in Table 4 shows that native urban residents having more than 10 local friends were more likely to be current smokers (b = 0.36, 95% CI = [0.11, 0.61], *p* < 0.01) than those with no local friends. Perceived neighborhood cohesion had no significant effect on tobacco use among either rural-to-urban migrants or native urban residents.

Table 5 presents the results of the relationship between local/trans-local ties and alcohol use. Model 5a showed that for rural-to-urban migrants, having more local friends were associated with higher frequency of drinking (b = 0.58, 95% [0.10, 1.06]; *p* < 0.05); this association remained marginally significant even when trans-local ties were included (Model 5b: b = 0.47, 95% CI = [−0.06, 0.99]; *p* = 0.08). Similarly, we observed that native urban residents with more local friends had significantly higher drinking frequencies (Model 6). Perceived neighborhood cohesion in the city and trans-local ties with migrants’ home communities were not associated with alcohol use. However, perceived neighborhood cohesion was negatively associated with drinking frequency among urban residents (b = −0.14, 95% CI = [−0.23, −0.06]; *p* < 0.01).

## 4. Discussion

While assimilating into the city and building local ties, rural-to-urban migrants in China maintain close social ties to their home communities. However, limited studies have examined the effects of local and trans-local ties on the health risk behaviors among migrant workers in China. Using nationally representative data, this study presented an overview and comparative analyses of the alcohol and tobacco use among rural-to-urban migrants and urban native residents and investigated how local and trans-local ties might affect substance use among the two populations in China. The results extended the research on social networks and health risk behaviors and provided evidence for further research and the development of public health policy on alcohol and tobacco use. We discuss the main findings below.

First, our findings revealed that rural-to-urban migrant workers had greater levels of tobacco and alcohol use than their urban counterparts after adjusting for sociodemographic variables. Such findings were not consistent with some previous studies showing that rural-to-urban migrants tended to report less substance use than urban residents [17,18,19]. However, these studies were mostly conducted in only one province in China and collected data more than 15 years ago (e.g., in 2004–2005 for [17,18]), which may not reflect current situations of substance use among rural-to-urban migrant workers in China. Based on the national-representative data from the 2012 Migrant Dynamics Monitoring Survey in China, one study found that the prevalence of migrants’ tobacco use was slightly lower than that in the general population [19]. However, the data of substance use in the general population they compared to were from another survey (i.e., 2010 Global Adult Tobacco Survey). Given the data collection of the two surveys was at different times and used different sampling methods and measures, results from such a comparison may not be conclusive. To understand the above substance use disparities between rural-to-urban migrants and urban natives, we could seek hints from prior studies on urban-rural inequalities in China. Modern alcohol and tobacco consumption have been consistently high in China since the 1980s (when China opened its door to the whole world). Among all countries, China continues to be the largest manufacturer and consumer of cigarettes in recent decades; drinking alcohol, as a cultural symbol of happiness/celebrity and recently as necessary skills for career advancement, has been even widely accepted in China for thousands of years, which is rarely seen in other countries [35,36]. Fortunately, with rapid modernization and social development, awareness of the negative health effects of alcohol and tobacco use has been largely improved among well-educated Chinese people. However, due to the long-term rural-urban division in China, rural areas are generally less developed and have lower levels of education. It is thus understandable that compared with urban elites, rural people have much less exposure to information about the harms of smoking and drinking [37]. Such lagged awareness might be partially attributable to the greater levels of rural-to-urban migrants’ tobacco and alcohol use since they are coming from rural areas. Future studies could directly examine the differential awareness of the substance use harm among rural peasants, migrant workers, and various social classes of urban natives.

Second, our findings showed that local and trans-local ties were differentially associated with rural-to-urban migrants’ tobacco and alcohol use. Specifically, more local friends were associated with more frequent drinking behaviors among rural-to-urban migrant workers, whereas the strength of trans-local ties was positively related to their tobacco use. The positive association between local social ties and alcohol use may reflect the adverse consequences of assimilating into the host society among rural-to-urban migrants in China. Chinese drinking culture encourages social drinking, and drinking alcohol is a means for individuals to establish and express relationships with one another [38]. Rural-to-urban migrants with more local friends tend to drink more frequently to cultivate “guanxi” (the Chinese expression of sentimental and instrumental interpersonal connections) and strengthen social connections in the receiving city. We also conjectured that a large network of local friends might indicate greater adherence to behavioral norms in cities that seem less restrictive of drinking. Studies have shown that urban residents have greater levels of alcohol consumption affordability and more access to drinking-related activities/events [39,40]. In this context, rural-to-urban migrants with more local friends may be subject to a higher level of social pressure to adopt group-specific normative health behaviors and to drink more frequently with their local contacts (e.g., to discuss work-related business or to strengthen “*guanxi*” in dining tables). In other words, although local ties might provide positive instrumental and emotional support for migrant workers, such ties could foster a type of negative assimilation and increase health risks among migrant workers. Both pros and cons of local ties need to be carefully examined in future research.

Additionally, it is worth noting that perceived neighborhood cohesion was associated with less frequent alcohol use only among urban residents, but not among the migrant workers. A growing body of research has suggested that higher neighborhood social cohesion is associated with better health and well-being outcomes. Neighborhood social cohesion may promote health and health behavior by distributing health information, providing social and psychological support, and reinforcing healthy norms that certain behaviors (e.g., not heavy drinking) are desirable. Our finding of the beneficial role of neighborhood cohesion in reducing alcohol use among urban residents was consistent with prior studies. However, the insignificant effect of neighborhood cohesion for rural migrants may be due to the residential segregation they experienced in cities [6]. Chinese internal migrants are often excluded from mainstream urban neighborhoods with a beautiful and clean environment, good public order, and harmonious neighborhood relationships. Instead, Chinese rural-to-urban migrants tend to reside in poor urban enclaves (e.g., urban villages) characterized by high population density, low level of regulation, unhealthy living environment, and frequent safety problems [41,42]. Despite the adverse environment of these migrant-concentrated neighborhoods, the majority of rural migrants do not want to move out because of the low living cost. To some extent, they are stuck in these enclaves that can provide limited resources to develop healthy behaviors. Future studies could conduct surveys and interviews in such migrant-concentrated neighborhoods to examine the unique effects of neighborhoods on migrant workers’ health and health behaviors.

Different from the analysis of alcohol use, we found that the number of past-year return visits to migrants’ sending areas (trans-local ties) was positively associated with their current smoker status, while the number of local ties was not a significant correlate. The positive relationship between the number of return visits and current smoker status was consistent with overseas evidence [26]. We interpreted the findings from two perspectives. First, visits back home were behavioral indicators of connections to home communities, which may reinforce adherence to social norms and health behaviors prevalent in the home communities. As the prevalence of tobacco use and tobacco abuse was higher among rural residents than urban residents in China [40,43], rural-to-urban migrants who went back to home communities more frequently were more likely to be exposed to the tobacco-promoting norms and context. Additionally, the price of cigarettes is generally lower in rural areas, so people who return to rural communities have more opportunities to purchase cheaper cigarettes and bring them back to cities. Second, maintaining trans-local ties in the sending communities (e.g., travel and gift expenses) may result in financial and emotional stress. Such stress may, in turn, increase the propensity for engaging in health risk behaviors. However, we did not have information about the specific reasons or detailed context for the return visits among rural-to-urban migrants (e.g., taking care of left-behind children or the death of a family member). Future research could further examine the reasons for the return visits and their associations with health risk behaviors.

Realizing the huge health burden (e.g., lung cancer and liver cancer) related to tobacco and alcohol use, the Chinese government has developed an ambitious action plan (“Health China 2030 Strategy”) and aimed to substantially reduce the prevalence of alcohol and tobacco use by 2030 [35,36,37,38,39]. However, due to the vast development gap between rural and urban areas, all tobacco and alcohol control/prevention measures progressed slowly for the rural population, including rural-to-urban migrants. Our findings suggested that we should spend immense efforts on promoting citizens’ awareness of the negative health effects of tobacco use, especially in rural China, since migrant tobacco users continued to be significantly influenced by their rural ties; for reducing their alcohol use, the receiving societies need to provide migrant workers more healthy access to establish and expand their urban local ties and fundamentally reduce their institutional social exclusion in urban areas. As latecomers of modernization, many other developing countries, similar to China, began developing their industrialization and urbanization rapidly in recent decades, leading to large-scale rural-to-urban migration within their countries. The models and results of the present research could inspire future cross-cultural studies to extend our understandings on the nexus between internal migration and health, plus providing more generalizable evidence for global policy shifts on how to improve migrant workers’ well-being.

Despite significant findings, this study was not without limitations. First, the study was cross-sectional, and causality could not be inferred from the results. Longitudinal investigations are warranted to elucidate whether social ties predict substance use among the migrant population. Second, we did not control for tobacco and alcohol use before migration in our models since such data were not available. More sophisticated measures on migrant workers’ prior tobacco and alcohol use thus need to be designed in future examinations. Third, we used self-report data of tobacco and alcohol use, which may be subject to certain reporting biases. Although the data were anonymous to reduce the potential inaccuracies, it will be helpful to adopt other measures (e.g., biochemical markers) to examine the criterion-related validity of self-report substance use data. Fourth, we included a single-item measure of current tobacco use status and drinking frequency. More comprehensive assessments of tobacco and alcohol use (e.g., quantity and duration of tobacco use/binge drinking) are needed to capture different types of substance use (e.g., light users vs. substance abusers). Fifth, we assessed trans-local ties using behavioral indicators of connectedness to the migrants’ sending areas. Although behavioral indicators of transnationalism were widely used and have been validated in previous studies [44], future studies may include additional indicators of trans-local ties, such as perceived connectedness to the home communities. Lastly, constrained by the lack of provincial-representative data, we did not conduct a provincial-level analysis on migrant workers’ tobacco and alcohol use, despite that we have controlled for the region in our analysis. Future studies may conduct a more comprehensive regional and provincial analysis on migrant workers’ tobacco and alcohol use, considering the great levels of internal inequality within China.

## 5. Conclusions

This study was a pioneering one which examined the roles of local and trans-local ties in health risk behaviors among rural-to-urban migrants, the largest disadvantaged social group in China. Based on a nationally representative survey of rural-to-urban migrants and their urban counterparts, our results revealed that migrant workers’ higher risks in terms of tobacco and alcohol use were embedded into their relationships with both sending societies and receiving societies. Among all rural-to-urban migrants, those with a larger local network tended to drink alcohol more frequently since they might form a risky lifestyle to actively socialize with their urban native contacts, aiming to overcome their social segregation and resource constraints experienced in urban areas. Migrant workers with stronger trans-local ties were more likely to be current tobacco users due to their continuing adoption of tobacco tolerance in their rural hometowns, which could be linked with the insufficient health knowledge and awareness in less developed rural China. To lower the health risks of these migrant workers, it calls for essential actions to reduce the exclusion they experience in urban areas and improve health-related education in rural areas.

## Figures and Tables

**Table 1:** Descriptive statistics.

	Full Sample (N = 7864)		Rural-to-Urban Migrants (N = 1426)		Urban Native Residents (N = 6438)		*t/chi*-Square Test
	Mean/%	SD	Mean/%	SD	Mean/%	SD	
Age, mean (SD)	42.79	−11.33	39.34	−11.25	43.56	−11.21	12.83 ***
Sex							
Male	53.08		52.1		53.29		0.66
Female	46.92		47.9		46.71		
Marital status							
Single	13.83		17.81		12.95		42.39 ***
Cohabitate	0.85		1.47		0.72		
Married	81.37		78.47		82.01		
Divorced/widowed	3.94		2.24		4.32		
Education							
Primary school or below	12.35		17.56		11.19		50.34 ***
Secondary school	52.74		62.99		50.47		
College or above	34.92		19.45		38.34		
Occupation							
Manufacturing industry	27.9		35.2		26.28		50.38 ***
Service industry	51.04		47.76		51.77		
Other	21.06		17.04		21.95		
Annual income (logged), mean (SD)	10.1	−2.49	10.1	−2.59	10.1	−2.47	−0.04
Region							
East	53.62		61.36		51.91		42.23 ***
Middle	21.43		18.3		22.12		
West	24.95		20.34		25.97		
Current tobacco use							
Yes	24.42		26.95		23.86		6.02 *
No	75.58		73.05		76.14		
Frequency of drinking							
Less than once per week	78.44		76.05		78.97		12.49 **
1–2 times per week	12.7		14.04		12.4		
3–4 times per week	4.28		3.86		4.37		
Almost everyday	4.58		6.04		4.26		
*Local ties*							
Number of local friends							
0	12.43		11.72		15.59		35.96 ***
1—5	40.36		39.75		43.09		
6—10	27.11		27.41		25.74		
>10	20.11		21.12		15.59		
Perceived neighborhood cohesion, mean (SD)	3.26	−0.81	2.93	−0.82	3.33	−0.79	17.11 ***
*Trans-local ties*							
Number of times going back to the home community, mean (SD)			5.6	−23.44			
Donation to the home community							
Yes			23.29				
No			76.71				
Help people in the home community							
Yes			37.48				
No			62.52				

* *p* < 0.05, ** *p* < 0.01, *** *p* < 0.001.

**Table 2:** The effect of migration status on tobacco use and alcohol use.

	Tobacco Use		Alcohol Use	
	Logit	[95% CI]	Logit	[95% CI]
Rural-to-urban migrants	0.19 *	[0.03, 0.35]	0.27 ***	[0.11, 0.42]
Age	0.01 ***	[0.01, 0.02]	0.02 ***	[0.01, 0.02]
Sex (ref: male)				
Female	−4.06 ***	[−4.34, −3.77]	−2.52 ***	[−2.70, −2.35]
Marital status (ref: single)				
Cohabitate	−0.05	[−0.73, 0.62]	0.19	[−0.44, 0.82]
Married	−0.02	[−0.23, 0.19]	0.06	[−0.14, 0.27]
Divorced/widowed	0.43 *	[0.04, 0.83]	0.33	[−0.03,0.69]
Education (ref: primary or below)				
Secondary	0.14	[−0.06, 0.35]	0.13	[−0.07, 0.33]
College or above	−0.52 ***	[−0.76, −0.29]	−0.10	[−0.32, 0.13]
Annual income (logged)	0.01	[−0.02, 0.04]	0.04 **	[0.01, 0.07]
Occupation (ref: other)				
Manufacturing industry	0.27 **	[0.10, 0.44]	0.19 *	[0.02, 0.35]
Service industry	0.19 *	[0.02, 0.36]	0.07	[−0.09, 0.24]
Region (ref: East)				
Middle	−0.09	[−0.25, 0.06]	0.03	[−0.12, 0.18]
West	0.11	[−0.04, 0.26]	0.21 **	[0.07, 0.35]

* *p* < 0.05, ** *p* < 0.01, *** *p* < 0.001.

**Table 3:** The association between sociodemographic variables and tobacco use and alcohol use among migrants and urban native residents.

	Current Tobacco Use				Frequency of Drinking			
	Model 1a Rural-to-Urban Migrants		Model 1b Urban Native Residents		Model 2a Rural-to-Urban Migrants		Model 2b Urban Native Residents	
	Logit	[95% CI]	Logit	[95% CI]	Logit	[95% CI]	Logit	[95% CI]
Age	0.01	[−0.01, 0.02]	0.01 ***	[0.01, 0.02]	0.01	[−0.01, 0.02]	0.02 ***	[0.01, 0.03]
Sex (ref: male)								
Female	−4.51 ***	[−5.24, −3.79]	−3.97 ***	[−4.28, −3.65]	−2.62 ***	[−3.01, −2.22]	−2.50 ***	[−2.70, −2.31]
Marital status (ref: single)								
Cohabitate	0.72	[−0.58, 2.02]	−0.40	[−1.22, 0.42]	0.80	[−0.24, 1.84]	−0.17	[−0.97, 0.63]
Married	−0.02	[−0.47, 0.43]	−0.02	[−0.26, 0.22]	0.09	[−0.33, 0.51]	0.06	[−0.18, 0.29]
Divorced/widowed	0.31	[−1.02, 1.64]	0.44 *	[0.02, 0.86]	−0.24	[−1.47, 0.99]	0.37	[−0.02, 0.76]
Education (ref: primary or below)								
Secondary	−0.21	[−0.65, 0.22]	0.25 *	[0.01, 0.49]	−0.13	[−0.52, 0.26]	0.21	[−0.02, 0.44]
College or above	−0.62 *	[−1.18, −0.06]	−0.45 **	[−0.71, −0.18]	−0.35	[−0.85, 0.16]	−0.02	[−0.28, 0.24]
Annual income (logged)	0.02	[−0.04, 0.09]	0.01	[−0.02, 0.04]	0.02	[−0.04, 0.08]	0.05 **	[0.01, 0.08]
Occupation (ref: other)								
Manufacturing industry	0.35	[−0.09, 0.79]	0.25 **	[0.06, 0.44]	0.15	[−0.26, 0.55]	0.17	[−0.01, 0.36]
Service industry	0.53 *	[0.09, 0.97]	0.12	[−0.06, 0.31]	0.05	[−0.35, 0.45]	0.06	[−0.12, 0.24]
Region (ref: East)								
Middle	−0.37	[−0.78, 0.03]	−0.04	[−0.21, 0.13]	−0.17	[−0.55, 0.21]	0.08	[−0.08, 0.24]
West	0.16	[−0.21, 0.53]	0.11	[−0.06, 0.27]	0.24	[−0.08, 0.57]	0.22 **	[0.06, 0.37]

* *p* < 0.05, ** *p* < 0.01, *** *p* < 0.001.

**Table 4:** The effect of local ties and trans-local ties on tobacco use.

	Rural-to-Urban Migrants				Native Urban Residents	
	Model 3a		Model 3b		Model 4	
	Logit	[95% CI]	Logit	[95% CI]	Logit	[95% CI]
*Local ties*						
Number of local friends (ref: =0)						
1—5	−0.03	[−0.48, 0.41]	−0.18	[−0.67, 0.31]	0.09	[−0.14, 0.32]
6—10	−0.24	[−0.72, 0.24]	−0.28	[−0.81, 0.24]	0.10	[−0.14, 0.34]
>10	0.30	[−0.22, 0.82]	0.12	[−0.46, 0.70]	0.36 **	[0.11, 0.61]
Perceived neighborhood cohesion	−0.00	[−0.19, 0.18]	0.04	[−0.16, 0.25]	0.00	[−0.09, 0.09]
*Trans-local ties*						
Number of times going back to the sending community			0.01 **	[0.00, 0.02]		
Donation to the sending communities (ref: no)						
Yes			−0.02	[−0.42, 0.39]		
Help people in the sending community (ref: no)						
Yes			0.15	[−0.21, 0.51]		

** *p* < 0.01. All models adjusted for sociodemographic variables, including age, sex, marital status, education, annual income, occupation, and region.

**Table 5:** The effect of local ties and trans-local ties on alcohol use.

	Rural-to-Urban Migrants				Native Urban Residents	
	Model 5a		Model 5b		Model 6	
	b	[95% CI]	b	[95% CI]	b	[95% CI]
*Local ties*						
# of local friends (ref: =0)						
1—5	0.18	[−0.24, 0.60]	0.06	[−0.40, 0.52]	0.18	[−0.05, 0.41]
6—10	0.06	[−0.40, 0.52]	0.01	[−0.49, 0.51]	0.43 ***	[0.19, 0.67]
>10	0.58 *	[0.10, 1.06]	0.47	[−0.06, 0.99]	0.54 ***	[0.29, 0.79]
Perceived community cohesion	−0.06	[−0.23, 0.11]	0.00	[−0.18, 0.19]	−0.14 **	[−0.23, −0.06]
*Trans-local ties*						
Number of times going back to the sending community			0.00	[−0.00, 0.01]		
Donation to the sending communities (ref: no)						
Yes			−0.09	[−0.45, 0.28]		
Help people in the sending community (ref: no)						
Yes			0.15	[−0.18, 0.48]		

* *p* < 0.05, ** *p* < 0.01, *** *p* < 0.001. All models adjusted for sociodemographic variables, including age, sex, marital status, education, annual income, occupation, and region.

## Data Availability

The overview and documents of the CLDS can be found at http://css.sysu.edu.cn/Data (accessed on 15 November 2021).
